# MGAE-DC: Predicting the synergistic effects of drug combinations through multi-channel graph autoencoders

**DOI:** 10.1371/journal.pcbi.1010951

**Published:** 2023-03-03

**Authors:** Peng Zhang, Shikui Tu

**Affiliations:** Department of Computer Science and Engineering, Center for Cognitive Machines and Computational Health (CMaCH), Shanghai Jiao Tong University, Shanghai, China; Tel Aviv University, ISRAEL

## Abstract

Accurate prediction of synergistic effects of drug combinations can reduce the experimental costs for drug development and facilitate the discovery of novel efficacious combination therapies for clinical studies. The drug combinations with high synergy scores are regarded as synergistic ones, while those with moderate or low synergy scores are additive or antagonistic ones. The existing methods usually exploit the synergy data from the aspect of synergistic drug combinations, paying little attention to the additive or antagonistic ones. Also, they usually do not leverage the common patterns of drug combinations across different cell lines. In this paper, we propose a multi-channel graph autoencoder (MGAE)-based method for predicting the synergistic effects of drug combinations (DC), and shortly denote it as MGAE-DC. A MGAE model is built to learn the drug embeddings by considering not only synergistic combinations but also additive and antagonistic ones as three input channels. The later two channels guide the model to explicitly characterize the features of non-synergistic combinations through an encoder-decoder learning process, and thus the drug embeddings become more discriminative between synergistic and non-synergistic combinations. In addition, an attention mechanism is incorporated to fuse each cell-line’s drug embeddings across various cell lines, and a common drug embedding is extracted to capture the invariant patterns by developing a set of cell-line shared decoders. The generalization performance of our model is further improved with the invariant patterns. With the cell-line specific and common drug embeddings, our method is extended to predict the synergy scores of drug combinations by a neural network module. Experiments on four benchmark datasets demonstrate that MGAE-DC consistently outperforms the state-of-the-art methods. In-depth literature survey is conducted to find that many drug combinations predicted by MGAE-DC are supported by previous experimental studies. The source code and data are available at https://github.com/yushenshashen/MGAE-DC.

## Author summary

Drug combination therapy is widely employed for various complex diseases because of its advantages of enhancing the efficiency, overcoming the drug resistance and reducing dose-dependent toxicity relative to monotherapy. To identify novel reliable and efficacious drug combinations for the patients, various methods have been proposed in the past decades. The trial-based methods are based on the clinical trials directly, but may cause patients to receive unnecessary or even harmful treatments. The experimental methods are labour and cost intensive, it is infeasible to test the complete drug combination space due to the combinatorial explosion. Computational methods provide attractive solutions to make the prediction based on the known data *in silico*, and thus narrow down the searching range of potential combinations. We have developed a novel deep learning method, named MGAE-DC, for predicting the synergistic effects of drug combinations. Our method explicitly characterizes not only synergistic combinations but also non-synergistic ones to obtain discriminative drug embeddings. Our method also learns common features of drug combinations across the cell lines for improved generalization performance. Computational experiments have verified the effectiveness of MGAE-DC.

This is a *PLOS Computational Biology* Methods paper.

## Introduction

Drug combination therapy, a treatment modality that combines two or more therapeutic agents, is a widely-used paradigm for various complex diseases such as cancer [1], hypertension [2] and infectious diseases [3]. Compared with the monotherapy, the drug combination therapy has the advantages of enhancing the efficiency, overcoming the drug resistance and reducing dose-dependent toxicity [4]. However, most of the drug combinations show additive effects which is equal to the sum of single-drug administrations, while rare drug combinations show synergistic effects or antagonistic effects where they have greater or lower effects than the sum of their individual administrations [5]. The drug combinations with strong synergistic effects, or synergistic drug combinations (SDCs), are attractive, new candidate therapies for clinical studies [6].

The effects of drug combinations are context-dependent. There is a pressing need to accurately identify synergistic effects of drug combinations for a given disease. Early studies for discovering novel SDCs are mainly based on clinical trials. The biggest problem for this kind of trial-based methods is that they may cause patients to receive unnecessary or even harmful treatments [7]. Although the experimental methods like high throughput screening technique can efficiently evaluate the synergy scores of many drug combinations across hundreds of cell lines and reduce the potential damage of trial-based methods, they are infeasible to test the complete drug combination space due to the combinatorial explosion [8]. Therefore, fast and efficient computational methods have become increasingly popular in identifying novel reliable and efficacious SDCs for clinical studies [9].

Recent methods for drug synergy prediction usually first construct informative features for drugs and cell lines, and then build prediction models over the features [10]. The quality of the extracted features for drugs and cell lines are critical to the prediction performance [11]. Early studies rely on hand-crafted features like drug molecular fingerprint, which is a numerical vector indicating the existence of drug substructures. For example, Sidorov et al. trained on the drug fingerprints a random forest (RF) and an extreme gradient boosting (XGBoost) model separately for each cell line, to predict the synergistic effects of drug combinations [12]. Besides, Preuer et al. and Kuru et al. both concatenated drug fingerprints and cell line genomic data as input features, used a normalization strategy to account for input data heterogeneity, and built conical layers to model the drug synergies [13, 14]. However, the prediction performances of these methods are limited by the design of hand-crafted features, which is labor-intensive and rely on the expert experiences.

To solve the above drawbacks and limitations, deep learning methods have been proposed [15]. For instance, Wang et al. developed DeepDDS which automatically captures features from drug chemical data and gene expression profiles to predict SDCs for given cancer cell lines [16]. In this model, the drug chemical structures were treated as graphs, and the drug features were learned by a graph convolutional network (GCN) which encodes molecular topology information efficiently. Considering cell lines as different relations, the synergy data of drug combinations were modeled as a relational GCN (R-GCN) by Zhang et al.’s SDCNet [17], where nodes are drugs, and edges are SDCs. Cell line-specific decoders were adopted to reconstruct the known SDCs, and predict new ones for each cell line, with the help of learned invariant features of drug combinations among the cell lines. Readers are referred to [9, 10] for a more comprehensive review.

The effects of drug combinations are context-dependent. Drug combinations may be synergistic in one cell line, but become additive or antagonistic in another one [18]. Meanwhile, the drug combinations may share invariant patterns across different cell lines [19]. The unique and common features of drug combinations both play indispensable roles in the drug synergy prediction. Besides, the existing methods are usually developed on the data of synergistic combinations, paying little attention to the non-synergistic ones, which limits their prediction performance.

In this paper, we propose a novel deep learning method, MGAE-DC, for predicting the synergistic effects of drug combinations across cell lines. Not only synergistic combinations but also additive or antagonistic ones are considered as multiple channels into a multi-channel graph autoencoder (MGAE) network. The representation learning of drug combinations becomes more discriminative between synergy and non-synergy than the existing methods because the non-synergistic combinations are explicitly modeled by our method. After obtaining the cell-line specific drug embeddings via MGAE, an attention mechanism is devised to fuse them across cell lines, and a common drug embedding is extracted to represent cell-line invariant features by cell-line shared decoders. Thus, both cell-line unique and common patterns are captured to improve the generalization performance across different cell lines. We evaluate the performance of MGAE-DC by comparing with state-of-the-art methods on four benchmark datasets. The comparison results, as well as ablation study, demonstrate the effectiveness of the proposed method in predicting the synergistic effects of drug combinations. Literature study indicate that the predictions by MGAE-DC are well supported by previously reported experiment data.

## Results

### MGAE-DC architecture

**Fig 1:**
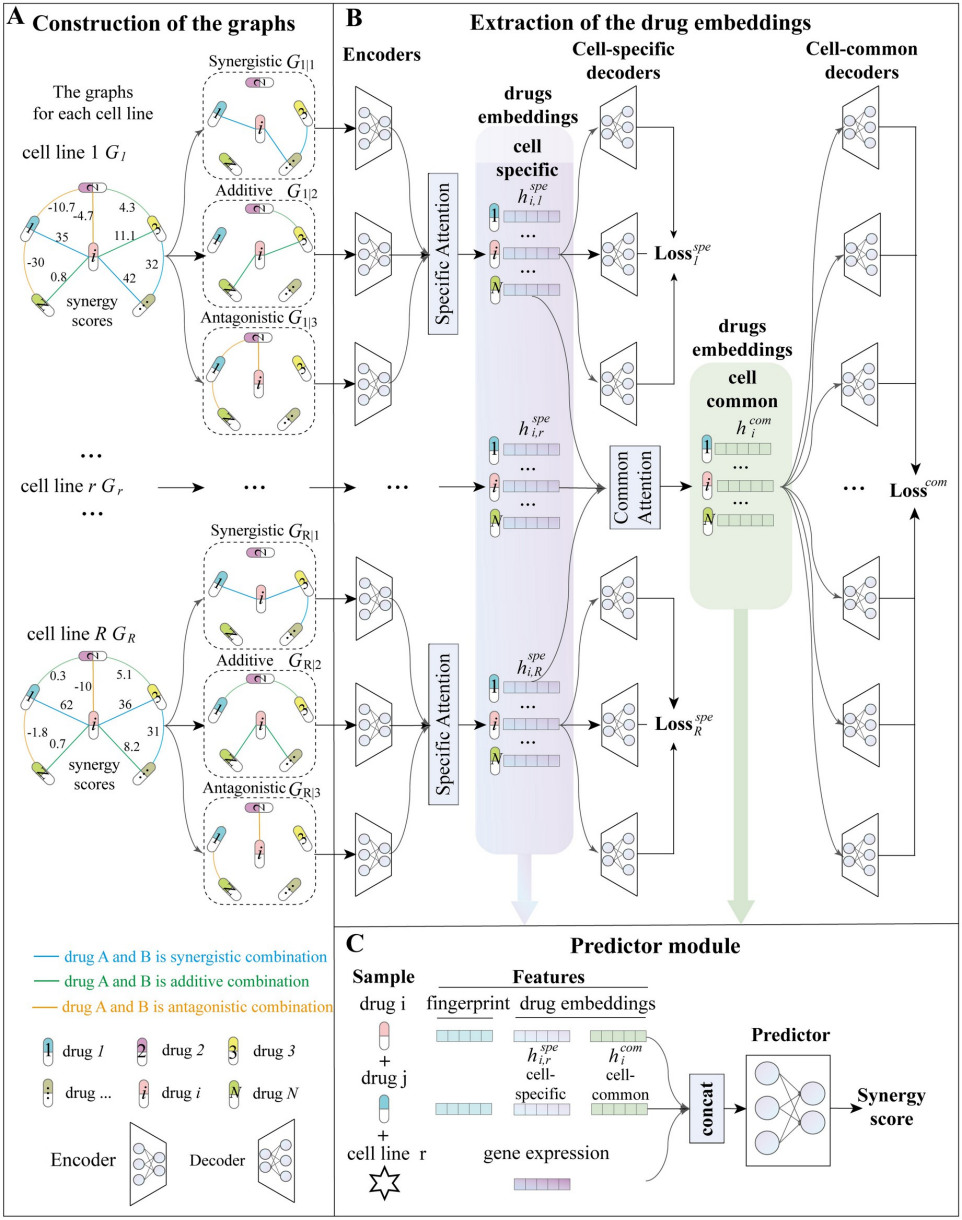
An overview of MGAE-DC.

MGAE-DC consists of an embedding module and a predictor module (Fig 1). The embedding module is implemented by a MGAE to learn low-dimensional drug embeddings. As given in Fig 1A, the synergy data of drug combinations in each cell line are represented as three graphs, i.e., synergistic graph, additive graph and antagonistic graph. The nodes are drugs, and the edges are determined according to the levels of their synergy scores, i.e., high, moderate, and low, for the three graphs respectively. Then, as in Fig 1B, MGAE is adopted for the graphs from a specific cell line to learn the features of combinations from the cell line. The obtained latent features are named as cell line-specific embeddings. An attention mechanism is developed to fuse the cell-line specific embeddings for each drug, and exploit the cell-line common patterns of combinations through partial parameter sharing in decoders. The model is trained by optimizing two types of reconstruction errors. One is on the three input graphs for every cell line, and the other is induced by cell-line common decoders to reconstruct the synergy data of all cell lines in one loss. Finally, in Fig 1C, a predictor module is built to train on the concatenated features of the learned drug embeddings, drug fingerprints, and cell line features, and to predict the synergy scores of drug combinations.

### Comparative results on all cell lines

We evaluate the performance of MGAE-DC in predicting the synergy scores of drug combinations on all cell lines, in comparisons with state-of-the-art methods. We adopt the stratified 10-fold cross-validation strategy. Table 1 summaries the performances of different methods on the O’Neil dataset under various synergy types. Our MGAE-DC method achieves the lowest RMSEs, i.e., 12.73, 4.15, 3.27 and 4.22 under synergy types Loewe, Bliss, ZIP and HSA, respectively. MGAE-DC also obtains the highest PCC under each synergy type. Furthermore, the results on the ALMANAC, CLOUD and FORCINA datasets are reported in S1–S3 Tables, respectively. The results consistently demonstrate that MGAE-DC outperforms other methods in most datasets.

**Table 1:** The synergy score regression performances on the O’Neil dataset, in terms of mean and standard deviation.

Method	MSE	RMSE	Confidence interval	PCC
**Loewe**				
EC-DFR	265.08 ± 32.78	16.25 ± 0.68	[224.38, 305.77]	0.71 ± 0.02
DeepSynergy	226.04 ± 11.12	15.03 ± 0.37	[212.24, 239.85]	0.75 ± 0.02
Matchmaker	223.35 ± 19.41	14.96 ± 0.65	[205.25, 253.44]	0.75 ± 0.03
SynPred	214.16 ± 11.66	14.73 ± 0.39	[196.86, 237.46]	0.76 ± 0.02
TranSynergy	208.73 ± 12.26	14.44 ± 0.32	[191.28, 221.19]	0.77 ± 0.02
HypergraphSynergy	184.14 ± 18.21	13.57 ± 0.72	[170.98, 197.31]	0.79 ± 0.02
PRODeepSyn	177.16 ± 16.52	13.32 ± 0.61	[156.65, 197.67]	0.81 ± 0.02
MGAE-DC	**162.21** ± **10.25**	**12.73** ± **0.43**	[**149.49, 174.93**]	**0.83** ± **0.01**
**Bliss**				
EC-DFR	30.37 ± 3.96	5.52 ± 0.36	[25.45, 35.28]	0.69 ± 0.01
DeepSynergy	29.31 ± 2.98	5.41 ± 0.28	[25.61, 33.04]	0.72 ± 0.05
Matchmaker	27.15 ± 3.41	5.26 ± 0.33	[22.92, 31.39]	0.73 ± 0.01
SynPred	26.64 ± 3.25	5.68 ± 0.29	[23.15, 32.58]	0.75 ± 0.03
TranSynergy	26.56 ± 3.29	5.62 ± 0.31	[23.04, 32.28]	0.75 ± 0.01
HypergraphSynergy	25.29 ± 4.16	5.01 ± 0.42	[18.61, 31.97]	0.77 ± 0.03
PRODeepSyn	18.78 ± 3.82	4.31 ± 0.44	[14.04, 23.52]	0.82 ± 0.02
MGAE-DC	**17.36** ± **3.17**	**4.15** ± **0.39**	[**13.42, 21.29**]	**0.84** ± **0.02**
**ZIP**				
EC-DFR	18.02 ± 0.57	4.24 ± 0.07	[17.32, 18.72]	0.74 ± 0.01
DeepSynergy	16.11 ± 0.86	4.01 ± 0.11	[15.04, 17.17]	0.77 ± 0.01
Matchmaker	16.21 ± 1.28	4.02 ± 0.16	[14.63, 17.82]	0.77 ± 0.02
SynPred	15.51 ± 0.46	3.93 ± 0.13	[14.24, 16.53]	0.78 ± 0.01
TranSynergy	15.48 ± 0.67	3.62 ± 0.16	[14.13, 16.86]	0.78 ± 0.02
HypergraphSynergy	13.52 ± 0.54	3.68 ± 0.06	[13.62, 15.29]	0.79 ± 0.02
PRODeepSyn	11.87 ± 0.17	3.45 ± 0.02	[11.66, 12.08]	0.83 ± 0.01
MGAE-DC	**10.68** ± **0.41**	**3.27** ± **0.06**	[**10.17, 11.18**]	**0.85** ± **0.01**
**HSA**				
EC-DFR	30.33 ± 4.82	5.49 ± 0.45	[24.35, 36.31]	0.70 ± 0.02
DeepSynergy	29.71 ± 2.41	5.45 ± 0.22	[26.71, 32.42]	0.71 ± 0.02
Matchmaker	27.62 ± 4.06	5.24 ± 0.44	[22.59, 32.66]	0.73 ± 0.02
SynPred	27.38 ± 3.22	5.21 ± 0.29	[22.38, 32.76]	0.73 ± 0.02
TranSynergy	27.14 ± 4.06	5.19 ± 0.32	[22.15, 32.62]	0.73 ± 0.02
HypergraphSynergy	26.11 ± 4.18	5.10 ± 0.41	[19.41, 32.97]	0.76 ± 0.02
PRODeepSyn	18.52 ± 2.24	4.30 ± 0.26	[15.74, 21.33]	**0.83** ± **0.01**
MGAE-DC	**17.89** ± **2.17**	**4.22** ± **0.26**	[**15.19, 20.59**]	**0.83** ± **0.01**

We further adopt leave-one-drug-out, leave-one-cell-line-out and leave-drug-pairs-out strategies, to comprehensively evaluate the generalization performance of our method on novel drugs, cell lines or drug pairs. S4 Table summaries the results on the O’Neil dataset using Loewe score. Consistent with the previous results, all methods achieve relatively low predictive performance when generalizing to novel drugs, cell lines or drug pairs [13, 20]. For the leave-one-drug-out strategy, EC-DFR achieves the lowest RMSE of 20.86, PRODeepSyn achieves the highest PCC of 0.46 and our method obtain the second best performance in terms of all metrics. For the strategy of leave-one-cell-line-out, MGAE-DC obtains the best performance in terms of RMSE, while MGAE-DC and DeepSynergy both achieve the highest PCC value of 0.57. Similarly, for the strategy of leave-drug-pairs-out, MGAE-DC obtains the lowest RMSE and the highest PCC with values of 17.8 and 0.7, respectively. The above results demonstrate the robustness of MGAE-DC when generalizing to novel cell lines or drug pairs.

Drug-drug synergy prediction has also been studied as a classification problem in the literature, i.e., predicting whether a drug combination is synergistic. For a comprehensive evaluation, we also modify MGAE-DC as a classifier and compare it with state-of-the-art classification models for this task. Table 2 reports the classification performances of different methods on the O’Neil dataset using Loewe score. Considering the high ratio of negative samples in the dataset, the evaluation metric AUPR is a relatively fair metric on imbalanced data, and it is taken as the primary metric here. MGAE-DC and PRODeepSyn both achieve the best performance in terms of AUPR with a value of 0.67. Besides, MGAE-DC achieves the best performance in terms of the ACC, F1 and Kappa with values of 0.95, 0.77 and 0.61, respectively. We conclude that MGAE-DC and PRODeepSyn are competitive methods on the classification task.

**Table 2:** The classification performances of different methods on drug-drug synergy prediction on the O’Neil dataset using Loewe score, in terms of mean and standard deviation.

Method	AUC	AUPR	ACC	F1	Kappa
EC-DFR	0.90 ± 0.01	0.57 ± 0.03	0.93 ± 0.01	0.61 ± 0.03	0.52 ± 0.04
DeepSynergy	0.90 ± 0.01	0.60 ± 0.04	0.93 ± 0.01	0.62 ± 0.05	0.56 ± 0.04
Matchmaker	0.91 ± 0.01	0.61 ± 0.03	0.93 ± 0.01	0.63 ± 0.05	0.56 ± 0.03
Jiang’s method	0.86 ± 0.03	0.45 ± 0.07	0.91 ± 0.01	0.63 ± 0.09	0.40 ± 0.08
SynPathy	0.86 ± 0.02	0.52 ± 0.03	0.92 ± 0.01	0.63 ± 0.05	0.52 ± 0.03
DeepDDS	0.86 ± 0.01	0.56 ± 0.03	0.92 ± 0.01	0.63 ± 0.05	0.44 ± 0.03
SynPred	0.89 ± 0.02	0.58 ± 0.03	0.93 ± 0.01	0.65 ± 0.04	0.45 ± 0.04
TranSynergy	0.89 ± 0.02	0.58 ± 0.03	0.93 ± 0.01	0.65 ± 0.04	0.48 ± 0.04
DTF	0.89 ± 0.01	0.58 ± 0.03	0.93 ± 0.01	0.65 ± 0.04	0.48 ± 0.04
SDCNet	0.90 ± 0.01	0.59 ± 0.05	0.93 ± 0.01	0.68 ± 0.06	0.57 ± 0.03
HypergraphSynergy	0.92 ± 0.02	0.63 ± 0.03	0.93 ± 0.01	0.68 ± 0.06	0.57 ± 0.03
PRODeepSyn	**0.94** ± **0.01**	**0.67** ± **0.02**	0.94 ± 0.01	0.71 ± 0.02	0.60 ± 0.02
MGAE-DC	**0.94** ± **0.01**	**0.67** ± **0.02**	**0.95** ± **0.01**	**0.77** ± **0.05**	**0.61** ± **0.01**

### Comparative results in each cell line

**Fig 2:**
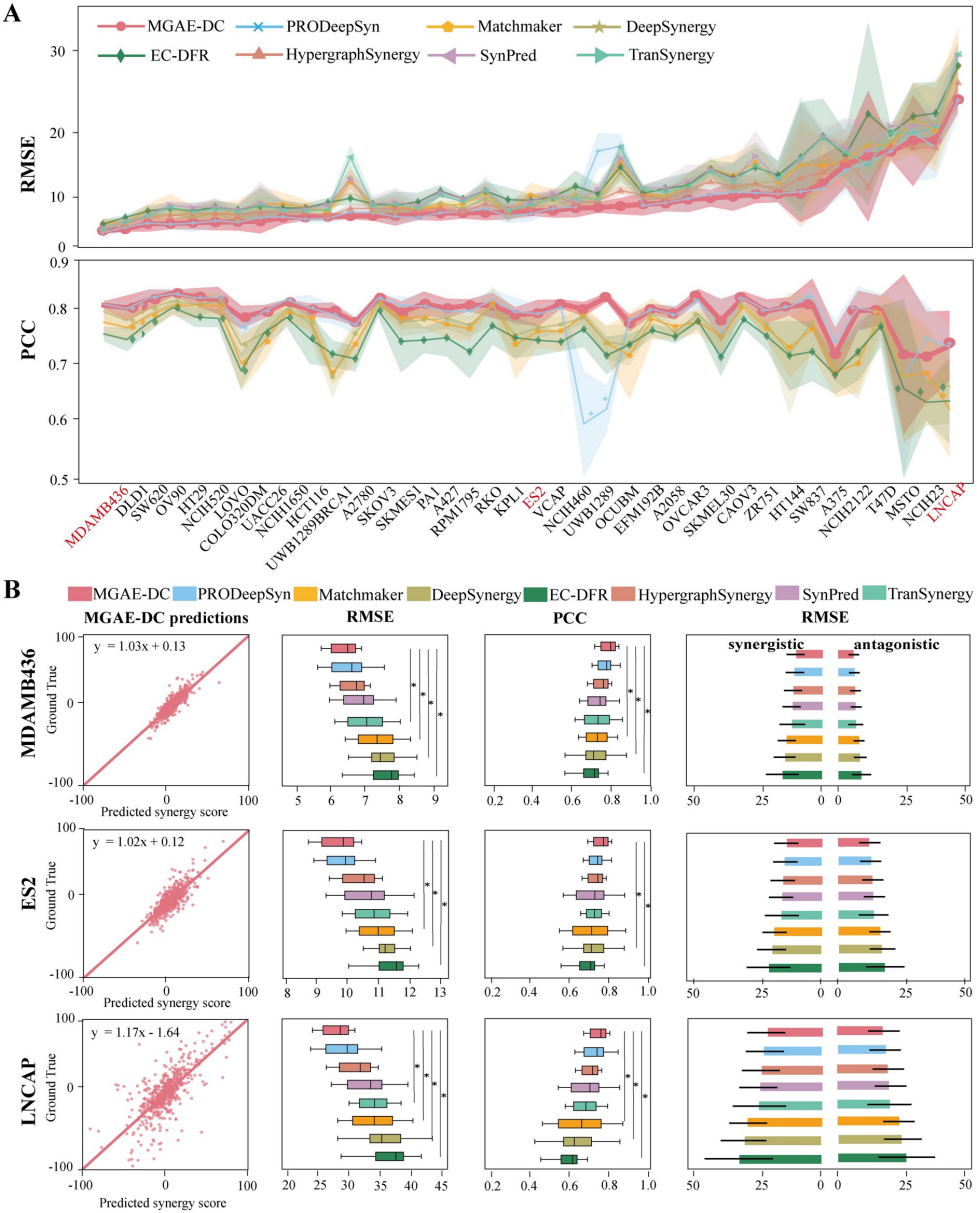
The performances of different methods in each cell line. (A) The RMSE and PCC of different methods in each cell line. The mean values of different folds are shown as solid lines. Error bars in terms of one standard deviation are shown as shaded areas. (B) The performances of different methods in the three representational cell lines. For each cell line, the scatter plot of MGAE-DC predicted synergy scores and the ground truth, the boxplots of RMSE and PCC of different methods, the RMSE of different methods in the synergistic and antagonistic drug combinations, respectively. The P-values are calculated by Wilcoxon signed-rank test. * represents P-value ≤ 0.05.

The synergistic effects of drug combinations are context-dependent. We evaluate the cell line level predictive performance of MGAE-DC on the O’Neil dataset with Loewe score. Fig 2A separately gives the distributions of RMSE and PCC of different methods in each cell line. MGAE-DC achieves consistent and robust performance in most cell lines, its minimum RMSE is 6.15 in cell line MDAMB436 and maximum PCC is 0.91 in cell line OVCAR3. Among the 39 cell lines, MGAE-DC achieves the lowest RMSE in 23 cell lines and the highest PCC in 27 cell lines, which outperforms other methods.

To display more details of the methods’ performances, three typical cell lines including MDAMB436, ES2 and LNCAP are selected, because different methods achieve superior, median and inferior level performances in these three cell lines in terms of RMSE, respectively (Fig 2B). In cell line MDAMB436, the scatter plot of MGAE-DC prediction results and the ground truth is displayed in the first column. The straight line in red, which represents the function between the predicted synergy scores and the ground truth fitted using the least squares regression, indicating their strong linear correlation. Then the distributions of RMSE and PCC of different methods in the cell line are shown in the second and third column, respectively. Similar performances are achieved by MGAE-DC and PRODeppSyn, and they significantly outperform other methods on all drug combinations from the corresponding cell line. Among the three types of drug combinations, additive combinations are the majority but less important, synergistic and antagonistic combinations are rare but more attractive candidates for clinical study. Therefore, we exclude the additive combinations, and further evaluate the performances of different methods on the synergistic and antagonistic combinations, respectively. The fourth column shows that MGAE-DC outperforms other methods on both synergistic and antagonistic combinations, which we are most concerned about. The methods in the other two cell lines ES2 and LINCAP achieve similar results and further demonstrate the effectiveness of MGAE-DC in predicting synergy effects of drug combinations in a specific cell line.

### Ablation study

We conduct an ablation study to investigate the contributions of the cell-line common drug embeddings, and the two input channels of additive and antagonistic drug combinations. Specifically, we evaluate and compare the performances of the following variants of MGAE-DC:

- MGAE-DC (FIN) is the variant of MGAE-DC that only uses the drug molecular fingerprint as drug features.
- MGAE-DC (SPE) is the variant of MGAE-DC that uses the drug molecular fingerprint and the corresponding cell line-specific embeddings as drug features.
- MGAE-DC (COM) is the variant of MGAE-DC that uses the drug molecular fingerprint and the corresponding cell line-common embeddings as drug features.
- MGAE-DC (SYN) is the variant of MGAE-DC that only uses the synergistic combinations to learn the drug embeddings.
- MGAE-DC (SYN+ADD) is the variant of MGAE-DC that uses the synergistic and additive combinations to learn the drug embeddings.
- MGAE-DC (SYN+ANT) is the variant of MGAE-DC that uses the synergistic and antagonistic combinations to learn the drug embeddings.
- MGAE-DC (CELL) is the variant of MGAE-DC that uses the low-dimensional cell line embeddings learned by Wang et al. [11] as cell line features instead of gene expression profiles.

Table 3 summaries the results of the ablation study on the O’Neil dataset using Loewe score. Compared with MGAE-DC (FIN), MGAE-DC achieves a lower RMSE and a higher PCC, indicating that the drug embeddings learned from the drug combinations’ synergy data are more accurate than molecular fingerprint features. Compared with MGAE-DC (SPE) and MGAE-DC (COM), the MGAE-DC also show superior performance. This demonstrates that cell line-specific and -common drug embeddings both facilitate the prediction results and they contain complementary information. When considering the additive or antagonistic combinations as additional two input channels, we observe that both channels benefit the training process and improve the prediction performance of the model trained on a single channel of synergistic drug combinations. Moreover, the antagonistic channel is more effective than the additive one. In addition, if we incorporate for cell lines the low-dimensional embeddings that integrate the PPI network with omics data by Wang et al. [11], MGAE-DC is further improved by MGAE-DC (CELL).

**Table 3:** The results of the ablation study.

Method	MSE	RMSE	Confidence interval	PCC
MGAE-DC (FIN)	179.71 ± 13.39	13.40 ± 0.50	[163.09, 196.34]	0.81 ± 0.02
MGAE-DC (SPE)	169.44 ± 13.8	13.01 ± 0.53	[152.31, 186.58]	0.82 ± 0.01
MGAE-DC (COM)	165.90 ± 7.16	12.88 ± 0.28	[157.01, 174.79]	0.82 ± 0.02
MGAE-DC (SYN)	166.56 ± 12.44	12.98 ± 0.53	[149.15, 178.98]	0.82 ± 0.02
MGAE-DC (SYN+ADD)	165.93 ± 8.09	12.92 ± 0.31	[156.82, 176.98]	0.82 ± 0.02
MGAE-DC (SYN+ANT)	164.57 ± 10.13	12.87 ± 0.39	[152.99, 178.15]	0.83 ± 0.02
MGAE-DC	162.21 ± 10.25	12.73 ± 0.40	[149.49, 174.93]	0.83 ± 0.01
MGAE-DC (CELL)	**157.13** ± **12.68**	**12.53** ± **0.50**	[**141.39, 172.88**]	**0.84** ± **0.02**

### Interpretation of the model

**Fig 3:**
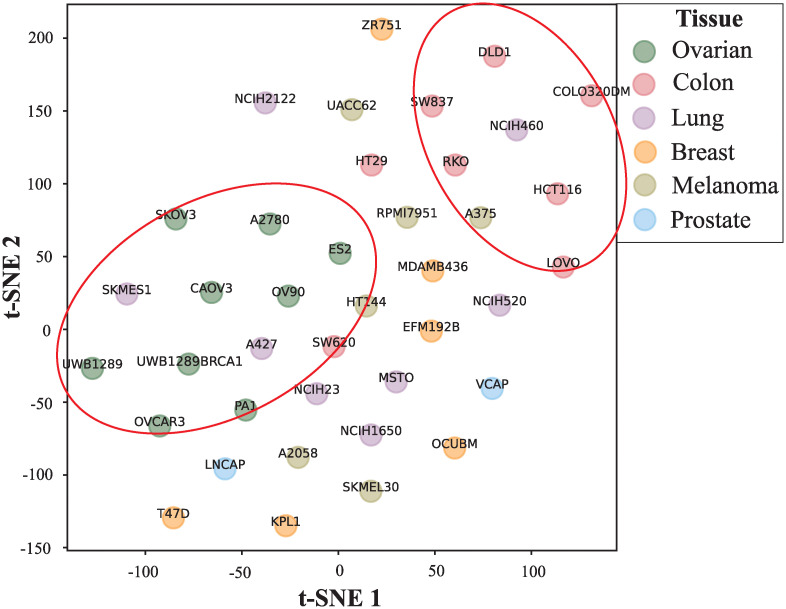
Visualization of drug Zolinza cell line-specific embeddings in 2-dimensional space using t-SNE.

Considering the drug embedding in specific cell line is critical in determining whether the drug combinations are synergistic on the cell line, we are curious about whether the model captures the cell line-specific features in the drug combinations’ synergy data. To visualize the cell line-specific drug embeddings, we mapped them into two-dimensional space with the first two components through the dimensionality reduction method t-SNE, respectively. Take the drug Zolinza for example (Fig 3), we found the embeddings in different cell lines belong to the same tissue are clustered together. Moreover, we find the Euclidean distance of many cell lines are similar with their synergy distances, which are consistent with that the same drug combination behave similarly on cell lines with similar embeddings. The embeddings of other drugs also show the same phenomenon (S1 Fig) and demonstrate that the model can capture the cell line-specific features in the drug combinations’ synergy data.

### The effect of different predictor

To evaluate the prediction performance of MGAE-DC more comprehensively, we change the NN to other predictors, such as Light gradient boosting machine (LightGBM), gradient boosting decision tree (GBDT), extreme gradient boosting (XGBoost), random forest (RF) and support vector regressor (SVR). Table 4 summaries the performances of models with different predictors on the O’Neil dataset with Loewe score. The best performance with the lowest RMSE of 12.73 and the highest PCC of 0.83 is achieved when using NN. One possible reason is that we integrate NN with batch normalization mechanism, which can effectively reduce the dependence of the NN model on initialized parameters, accelerate convergence and enhance generalization ability.

**Table 4:** The performances of different predictors for MGAE-DC.

Method	MSE	RMSE	Confidence interval	PCC
Neural Network	**162.21** ± **10.25**	**12.73** ± **0.4**	[**149.49, 174.93**]	**0.83** ± **0.01**
LightGBM	274.68 ± 32.84	16.55 ± 0.97	[233.92, 315.45]	0.69 ± 0.02
GBDT	274.73 ± 31.98	16.55 ± 0.94	[235.03, 314.43]	0.68 ± 0.02
XGBoost	279.41 ± 32.33	16.69 ± 0.95	[239.28, 319.54]	0.68 ± 0.02
Random Forest	286.95 ± 34.31	16.91 ± 0.99	[244.35, 329.55]	0.67 ± 0.02
SVR	347.59 ± 43.93	18.61 ± 1.15	[293.06, 402.13]	0.58 ± 0.03

### Case study

We further analyze the prediction results of MGAE-DC for the previously untested drug combinations based on the O’Neil dataset with Loewe score. In-depth literature survey was performed and we find many cases are consistent with previous studies. For example, vinorelbine and paclitaxel interfere with mitotic spindle function through different mechanisms of action, and their combination have been proved to show a synergistic activity in non-small cell lung cancer (NSCLC) [21, 22]. The prediction results of MGAE-DC for the combination in the three NSCLC cell lines including MSTO, NCIH23 and NCIH1650 are 33.49, 14.27, 10.34, respectively, indicating a synergistic potential in the treatment of NSCLC. Another example is that the combination of carboplatin and etoposide can be used to treat germ cell ovarian cancer [23], we check the prediction results of MGAE-DC for the ovarian cancer cell lines in our dataset namely CAOV3, OVCAR3, A2780, UWB1289BRCA1 and UWB1289, which are 86.28, 21.77, 9.97, 10.70 and 8.52, respectively. In addition, Lee et al. confirmed the synergistic effect of combination between etoposide and topotecan in ovarian cancer cell lines SKOV3 and A2780 through in vitro experiments [24]. MGAE-DC gives the prediction synergy scores of 44.27 and 10.34 in the two corresponding cell lines, respectively. Moreover, serial experiments have been conducted in cell line PA-1 for the combinations of paclitaxel with SN-38 [25], vinorelbine [26], 5-FU [27] and doxorubicin [28], respectively, and additive effects were found for all of these combinations. The prediction results given by MGAE-DC are consistent with these studies, and the predicted synergy scores are 1.14, -12.10, 3.62 and -10.22, respectively. In summary, the above in vivo and in intro experimental results all demonstrate the potential of MGAE-DC predicting novel reliable SDCs for clinical study.

## Discussion

In this paper, we propose a MGAE-based method, MGAE-DC, for predicting the synergistic effects of drug combinations. Our method considers the synergy data from the aspects of not only synergistic combinations but also additive and antagonistic ones, and integrating both unique and common features of drug combinations across different cell lines. Experiments on four benchmark datasets have demonstrated that MGAE-DC achieves consistent and robust performance and outperforms state-of-the-art methods. MGAE-DC is a valuable tool to facilitate the discovery of rational combination therapies for clinical study.

In the ablation study, we have demonstrated that the cell line-specific and -common drug embeddings contain complementary information and both are beneficial for the prediction performance. Moreover, the additive and antagonistic drug combination data have been explicitly incorporated and demonstrated to play a role complementary to the synergistic combination data by enhancing the discriminative capacity of the drug embeddings. Furthermore, we find that integrating the cell line embeddings, which learned from the PPI network and omics data, further improves the model’s prediction results, because the synergistic effects of drug combinations are context-dependent and accurate context embedding is definitely helpful.

MGAE-DC still has some shortcomings. In addition to the drug combinations’ synergy data, other data resources such as drug response data, drug-protein interactions and drug-disease interactions may also improve the model performance, which have not been included in MGAE-DC yet. For example, Jiang et al. has leveraged the drug combinations’ synergy data, drug-protein interactions and protein-protein interactions to do heterogeneous graph embedding for improving the model performance in specific cell lines [29]. Hence, incorporating more prior knowledge into the model to build more powerful and robust prediction models is the direction of our follow-up works. Besides, a drug combination with strong synergistic effect does not guarantee its effectiveness, and other characteristics like sensitivity and side effect are also critical to the clinical study [18]. Therefore, in addition to the synergistic effects of drug combinations, we are interested to develop models which is capable of simultaneously considering the combinations’ sensitivities and side effects when making the prediction.

## Materials and methods

### Data collection and preprocessing

The drug combinations’ synergy data are mainly comprised of four datasets including O’Neil, ALMANAC, CLOUD and FORCINA datasets [30]. Each drug combination in the datasets is represented by a drug-drug-cell line triple, and its synergistic effect is quantified by four synergy types namely Loewe additivity (Loewe), Bliss independence (Bliss), zero interaction potency (ZIP), and highest single agent (HSA), respectively. In general, combinations who with higher synergy scores are more synergistic, and vice versa [18]. A drug combination can be roughly classified into synergistic combination, additive combination, and antagonistic combination according to the thresholds in different synergy types. In particular, following the previous studies [31], the thresholds used are {0, 30} for the Loewe score, {-3.37, 3.68} for the Bliss score, {-3.02, 3.87} for the HSA score, and {-4.48, 2.64} for the ZIP score, where the combinations with scores higher than the large value are synergistic combinations, the combinations with scores lower than the small value are antagonistic combinations, and the other combinations are additive combinations. Table 5 summarizes the drug combinations’ synergy data in different datasets with various synergy types.

**Table 5:** Summary of the drug combinations’ synergy data for different datasets.

Synergy	Samples	O’Neil	ALMANAC	CLOUD	FORCINA
	Drugs	38	82	242	757
	Cell lines	39	59	1	1
	All combinations	22737	154596	29278	757
Loewe	Synergistic	1973	60	1430	67
	Additive	12087	31962	10688	525
	Antagonistic	8677	122574	17160	165
Bliss	Synergistic	7829	31470	5308	477
	Additive	10484	93585	3837	187
	Antagonistic	4424	29541	20133	93
ZIP	Synergistic	8806	29932	6058	504
	Additive	10803	90334	3500	138
	Antagonistic	3128	34330	19720	115
HSA	Synergistic	11958	21686	9078	479
	Additive	8567	91749	5211	150
	Antagonistic	2212	41161	14989	128

### The embedding module of MGAE-DC

#### Construction of the graphs in each cell line

The drug combinations’ synergy data in an arbitrary cell line *r* is represented as three graphs including synergistic graph *G*_*r*|1_ = (*V*, *E*), additive graph *G*_*r*|2_ = (*V*, *E*) and antagonistic graph *G*_*r*|3_ = (*V*, *E*) according to their synergy scores (Fig 1A), where *V* is the set of nodes (drugs), *E* is the set of edges in the corresponding graph. In particular, the adjacency matrixes of the graphs are represented as $A_{r|t}\in R^{N\times N},t=3$, where *N* denotes the number of drugs in all cell lines. The entries in the three adjacency matrixes *A*_*r*|1_(*i*, *j*), *A*_*r*|2_(*i*, *j*), *A*_*r*|3_(*i*, *j*) are set to 1 if the combination between drug *i* and drug *j* in cell line *r* is synergistic, additive, antagonistic, respectively; Otherwise, the entries are 0. Since the three graphs are undirected, we have *A*_*r*|*t*_(*i*, *j*) = *A*_*r*|*t*_(*j*, *i*).

#### MGAE for graphs in a specific cell line

MGAE is applied for the three graphs to extract the cell line-specific drug embeddings, which contain the unique features of combinations in a specific cell line (Fig 1B).

#### Encoder—Learning the cell line-specific drug embeddings

Taking the three graphs in an arbitrary cell line *r* and additional drug feature vectors as input, the encoder produces embeddings for the drugs. The three graphs are treated as a relational graph, where the relations refer to different types of graphs. The encoder assigns separate processing channels for each type of graph, then propagates and transforms information across different parts of the graph and across different graphs. Specifically, a single layer updating rule for drug *i* is defined by Eq (1):

$$\begin{array}{c} h_{i,r}^{\left(l+1\right)}=\sigma [\sum_{t=1}^{3}\sum_{j\in N_{i,r|t}}\frac{\alpha_{r|t}}{|N_{i,r|t}|}W_{r|t}^{\left(l\right)}h_{j,r}^{\left(l\right)}+W_{0,r}^{\left(l\right)}h_{i,r}^{\left(l\right)}], \end{array}$$ (1)

where $h_{i,r}^{\left(l\right)}\in R^{1\times d}$ is the embedding of drug *i* in cell line *r* at the *l*-th layer and *d* denotes the dimensionality of the drug embedding. $N_{i,r|t}$ denotes the set of the neighbor nodes of the drug *i* in the *t*-th graph. $|N_{i,r|t}|$ is the number of the neighbor nodes of the drug *i* in the corresponding graph and used as a normalization constant. *α*_*r*|*t*_ is a learnable parameter to weigh the contribution of drug *i*’s neighbor nodes from the *t*-th graph, which represents the graph level attention in a specific cell line, so we name it as specific attention. $W_{r|t}^{\left(l\right)}$ represents a trainable graph-specific weight matrix at the *l*-th layer, while $W_{0,r}^{\left(l\right)}$ is a weight matrix for drug *i* itself at the *l*-th layer. The function *σ*(*x*) is the ReLU activation function. With the above settings, we initialize the drug embedding $h_{i,r}^{\left(0\right)}$ with the corresponding drug molecular fingerprint.

Multiple layers are stacked to make the model more expressive and aware of the graph structure. Each layer contributes differently to the final prediction, therefore, an attention mechanism is employed to calculate the cell line-specific embedding for drug *i* in cell line *r* through:

$$\begin{array}{c} h_{i,r}^{spe}=\sum_{l=1}^{L}\;\beta_{r}^{\left(l\right)}\times h_{i,r}^{\left(l\right)}, \end{array}$$ (2)

where $\beta_{r}^{\left(l\right)}$ is a trainable parameter that indicates the attention weight of the *l*-th layer in cell line *r*, and *L* = 3 is the number of network layers.

#### Decoder

Three decoders are adopted to reconstruct the three graphs based on the cell line-specific drug embeddings learned by the encoder. For simplicity, the adjacency matrixes of the reconstructed synergistic graph, additive graph and antagonistic graph are denoted as $\hat{A_{r|t}}\in R^{N\times N},t=3$. In particular, the entry in the adjacency matrix of the reconstructed graph denotes a probability score indicating how likely the combination is connected. More precisely, utilizing the embedding vectors of drug *i* and drug *j* learned by the encoder, the adjacency matrix of the reconstructed graph is calculated through Eq (3):

$$\begin{array}{c} \hat{A_{r|t}}\left(i,j\right)=\sigma \left(h_{i,r}^{spe}D_{r|t}W_{r}D_{r|t}{\left(h_{j,r}^{spe}\right)}^{T}\right), \end{array}$$ (3)

where $W_{r}\in R^{d\times d}$ is a trainable weight matrix, which is shared in different graphs, modeling global interactions of drug combinations across different graphs in cell line *r*. $D_{r|t}\in R^{d\times d}$ is the diagonal matrix that capture the importance of each dimension in the drug embeddings $h_{i,r}^{spe}$ and $h_{j,r}^{spe}$ towards the *t*-th graph of cell line *r*. *σ* is the sigmoid function.

#### Loss function

To make the reconstructed graphs consistent with the original input graphs, we merge the mean square error (MSE) of the three graphs as the loss function. The MSE is calculated by Eq (4):

$$\begin{array}{c} Loss_{r}^{spe}=\sum_{t=1}^{3}\frac{1}{|y_{r|t}|}\sum_{\left(i,j\right)\in y_{r|t}}\|A_{r|t}\left(i,j\right)-\hat{A_{r|t}}\left(i,j\right){\|}^{2}, \end{array}$$ (4)

where (*i*, *j*) denotes the combination between drug *i* and drug *j* in cell line *r*, and *y*_*r*|*t*_ represents the set of combinations in the *t*-th graph. |*y*_*r*|*t*_| is the number of combinations in the *t*-th graph required to normalize the loss value.

#### MGAE for graphs in all cell lines

MGAE is applied for graphs in all cell lines to extract the cell line-common drug embeddings, which capture the common features of combinations among different cell lines.

#### Learning the cell line-common drug embeddings

The cell line-common drug embeddings are merged from the cell line-specific drug embeddings. Considering the embeddings from each cell line contribute differently to the prediction, we sort a cell line level attention mechanism, which we named common attention. More specifically, given the cell line-specific embedding of drug *i* $h_{i,r}^{spe}$ in all cell lines, its cell line-common embedding is calculated using Eq (5):

$$\begin{array}{c} h_{i}^{com}=\sum_{r=1}^{R}\;\alpha_{r}h_{i,r}^{spe}, \end{array}$$ (5)

where *R* is the number of cell lines, *α*_*r*_ is a trainable parameter that indicates the attention weight of drug embeddings from cell line *r*.

#### Decoder

3*R* decoders are adopted to reconstruct the graphs from all cell lines based on the cell line-common drug embeddings learned above. For simplicity, the adjacency matrixes of the reconstructed graphs in cell line *r* are denoted as $\hat{M_{r|t}}\in R^{N\times N},t=3$. In particular, utilizing the cell line-common embedding vectors of drug *i* and drug *j*, the adjacency matrix of the reconstructed graph is calculated through Eq (6):

$$\begin{array}{c} \hat{M_{r|t}}\left(i,j\right)=\sigma \left(h_{i}^{com}D_{r|t}WD_{r|t}{\left(h_{j}^{com}\right)}^{T}\right), \end{array}$$ (6)

where $W\in R^{d\times d}$ is a trainable weight matrix, which is shared in graphs from all cell lines, modeling global interactions of drug combinations across different graphs. $D_{r|t}\in R^{d\times d}$ is the diagonal matrix that capture the importance of each dimension in drug embeddings $h_{i}^{com}$ and $h_{j}^{com}$ towards the *t*-th graph in cell line *r*. *σ* is the sigmoid function.

#### Loss function

The MSE of graphs from all cell lines are merged as the loss function. The MSE is calculated by Eq (7):

$$\begin{array}{c} Loss^{com}=\sum_{r=1}^{R}\sum_{t=1}^{3}\frac{1}{|y_{r|t}|}\sum_{\left(i,j\right)\in y_{r|t}}\|A_{r|t}\left(i,j\right)-\hat{M_{r|t}}\left(i,j\right){\|}^{2}, \end{array}$$ (7)

where (*i*, *j*) denotes the combination between drug *i* and drug *j* in cell line *r*, and *y*_*r*|*t*_ represents the set of combinations in the *t*-th graph of cell line *r*. |*y*_*r*|*t*_| is the number of combinations in the *t*-th graph of cell line *r* required to normalize the loss value.

#### End-to-end training the embedding module

We deploy an end-to-end optimization approach training the embedding module to simultaneously learn low-dimensional cell line-specific and -common drug embeddings. The total loss is merged from the loss of models for each cell line and the loss of model for all cell lines, specifically, it is calculated by Eq (8):

$$\begin{array}{c} Loss=Loss^{com}+\sum_{r=1}^{R}\;Loss_{r}^{spe}, \end{array}$$ (8)

where *R* is the number of cell lines. All of the trainable parameters involved in the model are first initialized by the Xavier uniform initialization method, and jointly optimized via a gradient descent with the Adam optimizer. The model is implemented by TensorFlow (version 2).

### The predictor module of MGAE-DC

The predictor module receives the features of two drugs and one cell line to predict the synergy score of the drug combination. Fig 1C displays the architecture of predictor module, which is built by a triple-layers neural network with batch normalization. Three types of features are considered for each drug including its cell line-specific embeddings and cell line-common embeddings learned from the graph structured drug combinations’ synergy data in the embedding module, and the third type is the corresponding molecular fingerprint. The MSE loss function is used for training the predictor module.

### Methods for comparisons

To present the performance of MGAE-DC predicting the synergy scores of drug combinations, which is a regression task, we compare it with four advanced methods including DeepSynergy [13], Matchmaker [14], PRODeepSyn [11], EC-DFR [20], HypergraphSynergy [32], TranSynergy [33] and SynPred [34]. Since some existing methods treat the prediction as a classification task, we also compare the performance of MGAE-DC with these methods including DTF [23], DeepDDS [16], Jiang’s method [29], SynPathy [35] and SDCNet [17]. For the classification task, the synergistic drug combinations are labeled as positive samples while the other combinations are treated as negative samples. All existing methods are replicated using their publicly available programs. The detailed information of existing methods is summarized in S5 Table.

### Cross-validation strategies

**Fig 4:**
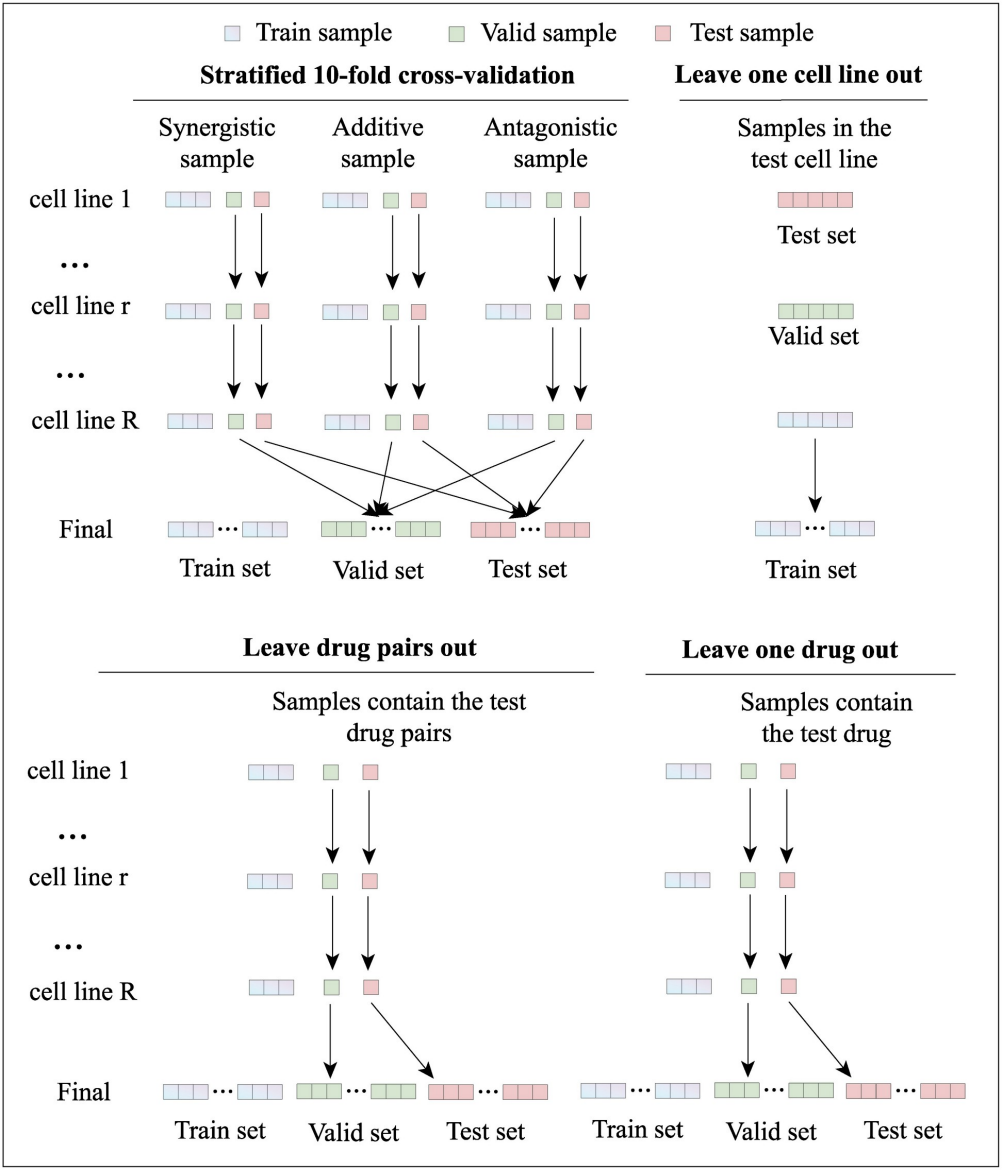
The schematics of different cross-validation strategies.

For experimental setup, we fist perform the stratified 10-fold cross-validation strategy to evaluate the performance of MGAE-DC (Fig 4). The synergistic drug combinations, additive drug combinations and antagonistic drug combinations are randomly split into ten equal-sized subsets in each cell line, respectively; one subset is selected from each type of combinations and the selected subsets from all cell lines are concatenated as the test set, while the remaining samples are taken as the training set. Then, to comprehensively evaluate the predictive and generalization performance, we further perform leave one drug out, leave one cell line out and leave drug pair out strategies (Fig 4). For the leave one cell line out, each cell line is regarded as the test cell line in turn, the samples in the test cell line are test set and the data in other cell lines form the training set. Similarly, the leave one drug out strategy treat each drug as test drug in turn and the samples contain the test drug are test set, the rest samples are training set. While the leave drug pairs out first randomly split the drug pairs into ten equal-sized subsets, then each subset is treated as test drug pairs in turn and the samples contain the test drug pairs are test set, the rest samples are training set.

### Hyperparameters selection

We consider different hyperparameter settings for the model including the dimensionality of the drug embeddings, learning rate, dropout rate and the hidden units in the predictor module (S6 Table). The best hyperparameters are determined by the grid-search and displayed in boldface.

### Evaluation metrics

The primary evaluation metrics for the regression task, which predicting the synergy scores of drug combinations, is MSE, the 95 confidence interval of MSE, root mean square error (RMSE) and the Pearson correlation coefficient (PCC). For the classification task, which predicting whether the drug combinations are SDCs, the commonly used evaluation metrics, including the area under the curve (AUC), accuracy (ACC), area under the precision recall (AUPR), precision, and the Cohen’s Kappa are used.

## Supporting information

S1 Fig Visualization of drug Dinaciclib and MK-4541 cell line-specific embeddings in 2-dimensional space using t-SNE. (TIF)

S1 Table The performances of different methods on the ALMANAC dataset. (XLSX)

S2 Table The performances of different methods on the CLOUD dataset. (XLSX)

S3 Table The performances of different methods on the FORCINA dataset. (XLSX)

S4 Table The performances of different methods using different cross-validation strategies. (XLSX)

S5 Table The detailed information of existing methods. (XLSX)

S6 Table Hyperparameters settings considered for MGAE-DC. (XLSX)
